# The Prognostic Value of Total Blood Count Parameter Ratios in Acute Pulmonary Embolism

**DOI:** 10.3390/diagnostics15010071

**Published:** 2024-12-30

**Authors:** Aynur Yurtseven, Kerem Ensarioğlu

**Affiliations:** 1 Department of Emergency Medicine, Ankara Etlik State Hospital, Ankara 06170, Turkey; aynuryurt7@gmail.com; 2 Department of Pulmonary Medicine, Faculty of Health Sciences Ankara Ataturk Sanatoryum Training and Research Hospital, Ankara 06290, Turkey

**Keywords:** pulmonary embolism, mortality, hospitalization, neutrophil-to-lymphocyte, platelet-to-lymphocyte, mean-platelet-volume-to-lymphocyte, ratio

## Abstract

**Background/Objectives:** Acute pulmonary embolism (PE) is a leading cause of cardiovascular mortality, characterized by nonspecific symptoms and variable clinical presentations. Accurate risk stratification is essential for effective management. While conventional tools like the simplified pulmonary embolism severity index (sPESI) and imaging modalities are widely used, they are often costly and have limitations in predictive accuracy. Inflammatory and coagulative markers, such as the neutrophil-to-lymphocyte ratio (NLR), platelet-to-lymphocyte ratio (PLR), and mean-platelet-volume-to-lymphocyte ratio (MPVLR), have shown promise in thrombotic conditions. This study explores their prognostic value in PE, focusing on their associations with risk stratification and clinical outcomes. **Methods:** This retrospective study included 231 adult patients diagnosed with PE at a tertiary care center. Exclusion criteria included recent infection, autoimmune diseases, or immunosuppressive therapy. Laboratory data, clinical parameters, and outcomes (e.g., hospitalization duration, complications, and mortality) were analyzed. Ratios were calculated from routine blood counts, and statistical comparisons were conducted between low- and high-risk groups based on sPESI. **Results:** High-risk patients (*n* = 203) exhibited significantly higher troponin, blood urea nitrogen, aspartate aminotransferase, lactate, the NLR (median 4.9 vs. 2.7, *p* = 0.005), and the MPVLR (median 7.1 vs. 3.9, *p* = 0.001) compared to low-risk patients. The PLR showed no significant difference between risk groups (*p* = 0.233). An elevated NLR, PLR, and MPVLR correlated with ICU admission, intubation, and mortality (*p* < 0.001, *p* < 0.007, and *p* < 0.001, respectively). The NLR was the most consistently associated with hospitalization duration and mortality, while the MPVLR and PLR were less predictive of overall hospitalization. **Conclusions:** The NLR, MPVLR, and PLR are cost-effective, easily calculable markers with the potential for improving risk stratification in PE patients. Among these, the NLR showed the strongest prognostic value, correlating with multiple clinical outcomes. Multicenter studies are needed to validate these findings further and establish clinical utility.

## 1. Introduction

Acute pulmonary embolism (PE) is the third leading cardiovascular mortality cause after acute myocardial infarction (AMI) and stroke [1]. In the United States of America, PE is reported to cause between 100,000 and 200,000 deaths yearly [2]. PE may present with a varying range of clinical findings and nonspecific symptoms. Appropriate timed risk stratification and early diagnosis remain the cornerstones of PE management. The pulmonary embolism severity index (PESI), echocardiography, lower extremity venous doppler ultrasonography, and computed tomography pulmonary angiography (CTPA) have been used along with clinical judgment and vital signs for the diagnostic process and risk evaluation among PE patients and those suspected with PE [3,4,5]. These tests and evaluation methods often suffer from low positive predictive value and are cost-intensive, which necessitates further studies for other possible markers for risk assessment [6].

Currently, studies in PE pathology have stated that inflammation caused by neutrophils and macrophages was present in pulmonary arteries and the right ventricle and suggested a correlation between inflammatory and coagulative processes that might further exacerbate PE and other thromboembolic events [7,8,9]. Similarly, among patients with pulmonary hypertension (PHT), pro-inflammatory and coagulative processes were attributed to increased microparticle creation by thrombocyte, leukocyte, and activated endothelial cells [10].

In this literature review, increased mean platelet volume (MPV) is observed to be a component of a higher thrombosis presence. Especially in coronary heart disease, AMI, restenosis after percutaneous coronary intervention, ischemic cerebrovascular events, venous thromboembolism, and MPV and its ratio to lymphocyte count (MPVLR) have been reported as predictors of thrombosis and could be used as a promising marker for mortality prediction [11,12,13].

In this study, we aimed to investigate the role of the MPVLR, which was not utilized before, in evaluating PE patients’ prognosis and mortality, in addition to the neutrophile-to-lymphocyte ratio (NLR) and platelet-to-lymphocyte (PLR) ratio.

## 2. Materials and Methods

This study was conducted at the emergency department of a tertiary state hospital, which has around 750,000 yearly patient admissions and an inpatient capacity of 4050 patients. After approval from the hospital’s local ethics committee (approval number AESH-BADEK-2024-523; approval date 10 July 2024), patients evaluated at the emergency department for PE between the dates of 1 January 2023 and 1 January 2024 were investigated retrospectively. The inclusion criteria of this study were being over 18 years old and having a PE diagnosis confirmed within the emergency service by a CTPA. Patients younger than 18 years old, those with an infection history within the last two weeks regardless of the origin, autoimmune disease presence, and those under immunosuppressive treatment with the immunosuppressive definition being designed as having a treatment regimen of 20 mg methylprednisolone daily or more equivalent treatment were excluded.

The patients’ demographic characteristics, laboratory results, simplified PESI (sPESI) score, hospitalization duration, one-month complication, and mortality were recorded from the hospital’s electronic record system, which was integrated into the national patient record system. Regarding ratio evaluation, NLR was calculated by dividing absolute neutrophile count to absolute lymphocyte count in peripherally sampled total blood count, which was requested as a part of the routine evaluation for PE patients. Similar calculations were performed for the PLR and MPVLR, with their respective values of platelet count and mean platelet volume to lymphocyte count.

Additional parameters that were included in this study were liver and renal function tests, which included aspartate aminotransferase (AST), alanine transaminase (ALT), blood urine nitrogen, creatinine, and lactate from venous sampling. The reports confirming PE diagnosis were made by specialist radiologists. Additional demographic information, including smoking and alcohol history and comorbidities that were not accepted as a factor for affecting overall immune system response, as stated in the exclusion criteria, were not recorded.

The patients’ results were put into a Microsoft Excel file for overall evaluation. After investigating any mis-inputs and values, the data were moved to a statistics module (IBM Version 25th for Windows). The initial assessment was performed by descriptive analysis, for which values were given with means and standard deviations or with medians and percentiles as required. Parametric distribution was evaluated using a Q-Q plot analysis. Chi-square tests were utilized to compare nominal variables. Depending on the parametric or nonparametric distribution, the comparisons of groups were made using independent samples of the *t*-test or Mann–Whitney U test. Spearman’s rho was utilized for correlation analysis for nonparametrically distributed groups.

*p* values at or below 0.05 were accepted as statistically significant. The study sample was calculated by the assumption that at least 200 patients were required to differentiate between two groups, with a type 1 error of %5, an allocation of 1 to 4, in favor of either group, and a power of 80%.

## 3. Results

A total of 285 patients were investigated for this study. Twenty-one patients were excluded from the initial evaluation due to inadequate available records or patients refusing to share the records. Fourteen patients had an infection history within the last two weeks, five patients had been diagnosed with PE before emergency admission, six patients had a history of autoimmune disease, and excluding these six patients, eight patients had an immunosuppressive treatment history. The remaining 231 patients were included in this study. The gender distribution was nearly equal, with 113 female patients (48.9%) and 118 male patients (51.1%). The mean age was 67 (±15) years old. When patients were divided into two groups according to the risk stratification of sPESI, the majority of the patients (n = 203) were in the high-risk group. In the comparison between groups, there was a statistical difference between age (59 to 68 years old, *p*-value of 0.004), troponin levels (a median of 6.1 ng/L to 29.8 ng/L, *p*-value of 0.001), blood urea nitrogen (a median of 34.7 mg/dL to 38.8 mg/dL, *p*-value of 0.025), aspartate aminotransferase (a median to 19 U/L to 25 U/L, *p*-value of 0.04) and lactate (a median of 1.57 mmol/L to 1.98 mmol/L, *p*-value of 0.032) between low- and high-risk groups, respectively. Other parameters requested in the routine renal and kidney function tests did not vary between groups (Table 1).

Regarding total blood count, the low-risk group patients had an overall lower median lymphocyte count (a mean of 2.56 μ/L to 1.67 μ/L, *p*-value of 0.001) than the high-risk group. A mortality analysis could not be given due to the meager mortality count (n = 2, 7.1%) observed in the low-risk group. However, the high-risk group had a higher mortality count (n = 39.2, 19.2%) (Table 1).

Regarding PTE severity and hospitalization evaluation, it was observed that all patients had been hospitalized at least once during the initial period of PE diagnosis. Patients in the high-risk group had a higher PE severity (n = 16, 7.9%) and a higher requirement for intensive care unit (ICU) admission (n = 77, 37.9%) compared to the low-risk group (n = 1, 3.6%, and n = 4, 14.3%, respectively); however, due to low patient count in the low-risk group, a statistical evaluation could not be stated. Intubation requirement and pulmonary arterial involvement did not vary between groups. The total hospitalization (a median of 10 days to 7 days, *p*-value 0.007) and ward admission duration (a median of 8 days to 6 days, *p*-value 0.006) were longer among patients in the high-risk group compared to the low-risk group. The ICU admission duration did not vary between groups. The neutrophile-to-lymphocyte ratio (NLR) was higher in the high-risk group (a median of 4.9 to 2.7, *p*-value 0.005), and similarly, the mean-platelet-volume-to-lymphocyte ratio (MPVLR) was also higher in the high-risk group (a median of 7.1 to 3.9, *p*-value 0.001). The platelet-to-lymphocyte ratio (PLT) had no statistical difference between groups (a median of 156 to 143.3, *p*-value 0.233) (Table 2)

The ratios were then evaluated according to subgroups of PE severity, ICU admission requirement, intubation requirement, and overall mortality. None of the ratios had a statistical difference regarding PE severity. The NLR, PLR, and MPVLR were higher in the patients requiring ICU admission (*p*-value of 0.001 for all groups). Similar observations were made for intubation requirements, as patients requiring intubation had overall higher scores (*p* values of 0.025, 0.008, and 0.011 for the NLR, PLR, and MPVLR, respectively). Regarding mortality, survivors had overall lower values for the NLR, PLR, and MPVLR compared to the patients lost during this study (*p* values of 0.001, 0.007, and 0.001, respectively) (Table 3).

In the correlation analysis performed with the ratios and hospitalization durations, no parameters were found to be correlated with ward admission (*p* values of 0.285, 0.325, and 0.388 for the NLR, PLR, and MPVLR, respectively). Only the NLR correlated with ICU admission duration (*p*: 0.025; correlation coefficient of 0.248). When evaluated for total hospitalization duration, the NLR and PLR had a positive, albeit weak, correlation with the total duration (*p*: 0.153, correlation coefficient 0.153, and *p*: 0.028, correlation coefficient 0.145, respectively) (Table 4).

## 4. Discussion

In this study, when patients were evaluated according to their PE risk scoring, the overall distribution and results, such as a higher troponin and lactate levels in the high-risk group, were expected. The hospitalization duration was also distributed according to the severity, with a higher hospitalization duration for the higher-risk group. For ratio evaluation, the NLR and MPVLR were higher in the high-risk group, while the PLR did not vary between groups. Despite this, all three were correlated with intubation and mortality.

When investigated for hospitalization duration, the NLR was associated with ICU and total hospitalization duration, while the PLR was only correlated with total duration. Overall, it could be stated that the NLR, as a parameter in patients admitted with PE, had changed between the low- and high-risk groups, was correlated with ICU duration, intubation, and mortality, and out of the three ratios, was the one with the most statistically significant factors. The MPVLR, while similar to the PLR in terms of mortality and intubation, did not have any significance regarding hospitalization duration. It was only superior in the observation that, unlike the PLR, the MPVLR did change between the low- and high-risk groups enough to be statistically significant. As such, while reliable according to risk evaluation, the MPVLR did not affect the hospitalization for PE. The PLR could be considered the middle ground between the NLR and MPVLR, as while it did not vary regarding risk classification, it was similar to the NLR except for not correlating with ICU hospitalization duration.

It was established that thrombocytes play an essential role in atherosclerosis and thromboembolic events. Similarly, upon activation, endothelial cells release chemokines that activate monocyte and neutrophil migration and thus contribute to plaque production [14]. As a definition of thrombocyte size, MPV could also be considered a predictor of thrombocyte activation [15]. Thrombocytes of increased size have been shown to produce more vasoactive and prothrombotic factors (including thromboxane A2, serotonin, and adenosine triphosphate) and aggregate at a faster pace, which leads to a more prominent reaction and eventually leads to an increased thrombosis state [16,17,18]. Overall, MPV could be used as a prognostic factor in thrombosis presence and severity.

A meta-analysis performed in 2017 regarding MPV’s role in patients with PE did not report any differences in MPV between patients diagnosed with PE and control groups [19]. Later studies, however, had reported a significant increase in MPV in patients with newly diagnosed and reoccurring PE [20,21]. This was supported by another meta-analysis performed by Lin et al., in which 205 studies were evaluated, and a higher MPV was reported to be associated with PE [22]. This was supported by another meta-analysis performed in 2020, which showed that PE was correlated with an increase in MPV. However, MPV could not be reliably utilized for risk assessment and classification [23]. It can be said that studies in the literature currently vary in the estimation of MPV’s role in PE, which can be attributed to patient selection and the severity of PE among them. Another cause that might have affected these studies could be PE’s origin and contributing risk factors. Our study tried to overcome one of these issues by evaluating patients according to the risk stratification and evaluation of these subgroups. Supporting the mentioned studies, our study did not reveal a difference in MPV between low-risk and high-risk PE patients.

A combination of other parameters with MPV could be utilized as a new method of investigating thrombosis burden. In the literature, a higher MPVLR, especially in diabetic patients diagnosed with non-ST elevation myocardial infarction, could be used as an independent risk factor for early and late mortality [24]. Niu et al., in a retrospective study of 643 patients with total coronary occlusion, reported that the MPVLR was markedly elevated [25]. Our study reported similar findings, as patients in the high-risk PE group had a higher MPVLR compared to those in the low-risk group.

Regarding other parameters, the elevated BUN levels in the high-risk group were comparable with the findings presented in the literature. In patients with acute PE, reduced renal perfusion and right ventricular diastolic dysfunction contribute to increased BUN. Renal function tests have also been known to correlate with dysregulation in microcirculation and were reported as an additional method of PE severity evaluation to non-invasive blood pressure monitorization [26]. Despite BUN being elevated in the high-risk group, both risk groups had similar creatinine levels. These correlations with a higher baseline BUN level and increased risk of a higher PE stratification have been reported in the literature, with Jenab et al. stating that BUN levels were an independent marker of mortality among PE patients, even for those within the same risk group [27].

As for cardiac biomarkers, troponin levels were found to be increased in high-risk groups, which could be attributed to myocardial cell damage and right ventricular dysfunction, which was compatible with other studies in the literature. Integration of these biomarkers, not only as a component of right ventricular dysfunction but also as an independent risk factor, has also been debated, with the currently accepted view being that they are not capable of identifying patients’ risk in an isolated manner, and thus, a combined clinical approach is still required to access overall cardiac function [28].

The NLR and PLR are other ratios that have been used in addition to the MPVLR in PE evaluation. Bi et al. reported in their observative study that the NLR was lower in the survivors of PE, compared to those lost in follow-up [29]. Telo et al. had stated that in addition to the NLR, the PLR was also increased in high-risk PE patients [30]. Kundi et al.’s study also supported the increase in the PLR among high-risk PE patients [31]. Our study did not show a difference in risk group regarding the PLR; however, we agreed that the PLR and MPVLR were statistically different in low- and high-risk groups. While all three parameters did not correlate with PE severity in correlation analyses, the correlation was present regarding mortality, ICU admission, and intubation requirements, thus supporting the overall statement of the mentioned ratios playing a role in PE severity evaluation. The variance in studies present in the literature, including our study’s results, could be mainly explained by the presence of other factors, mainly of an infectious nature, affecting the total blood count, despite the patient selection criteria’s role in reducing this effect. Regardless of the results, the main stance of the literature is similar to what we had reported, as while the mentioned ratios as stand-alone markers do play a role, utilization of them as a group and/or with other parameters remains the more supported option [32].

This study’s main limitation was that it was performed as a single-center study. While comprehensive in nature regarding ratio evaluation, due to the center being a tertiary one, the majority of the patients were evaluated in the high-risk group; thus, increasing centers, mainly in secondary care, could have provided more patients to the low-risk group. This aspect should be kept in mind in possible further studies, as evaluating patients in more stable conditions may give different results. This limitation could also be extended to patient selection, as patients who had to be evaluated in emergency care were included as per this study’s design. Stable patients who were incidentally diagnosed in outpatient care or patients deemed suitable enough to be followed in outpatient care by pulmonologists could also affect the results, similar to the hospital selection factor mentioned earlier. This study’s other limitation was relatively limited information availability regarding other comorbidities, such as smoking and alcohol history, as these assessments were not included in the emergency pulmonary consultation evaluation and, as per this study’s design, were not recorded unless comorbidity was assumed to be a risk factor for immune system response, in which case the patient had already been excluded. While the inclusion of all comorbidities might have been more informative, considering the exclusion criteria, we assume the evaluation would not have changed.

## 5. Conclusions

In conclusion, the MPVLR, NLR, and PLR are biomarkers that could be utilized in PE evaluation. Because total blood count is routinely requested in patients with PE, these biomarkers are also cost-effective and easy to calculate.

## Figures and Tables

**Table 1:** Demographic characteristics, admission laboratory values, and outcome of patients with risk classification.

Parameters (Median, 25th–75th)		sPESI Classification			*p* Value
		Low-Risk Group (n = 28)	High-Risk Group (n = 203)	Total (n = 231)	
Gender (%)	Male	13 (46.4)	105 (51.7)	118 (51.1)	0.599
	Female	15 (53.6)	98 (48.3)	113 (48.9)	
Age (years, ±SD)		59 (16)	68 (15)	67 (15)	0.004
Troponin (ng/L)		6.1 (2.5–15)	29.8 (11.3–65)	24.8 (7.9–59.3)	0.001
Glucose (mg/dL)		129 (99–233)	123 (104–173)	123 (104–176)	0.897
Blood Urea Nitrogen (mg/dL)		34.7 (24.8–39.3)	38.8 (28.0–55.5)	38 (28–54)	0.025
Creatinine (mg/dL)		0.97 (0.85–1.10)	0.95 (0.76–1.27)	0.95 (0.77–1.27)	0.981
AST (U/L)		19 (16–25)	25 (17–64)	24 (17–33)	0.04
ALT (U/L)		16 (11–23)	17 (11–26)	20 (11–26)	0.963
Lactate (mmol/L)		1.57 (1.24–2.42)	1.98 (1.50–2.94)	1.9 (1.41–2.9)	0.032
D-Dimer (μ/mL)		3.10 (1.15–4.61)	2.6 (1.51–5.48)	2.6 (1.42–5.20)	0.693
Total Blood Count (±SD)					
White Blood Cell (μ/L)		11.14 (2.95)	10.63 (3.85)	10.69 (3.75)	0.495
Hemoglobin (g/dL)		13.0 (2.5)	12.4 (2.3)	12.5 (2.3)	0.187
Lymphocytes (μ/L)		2.56 (1.1)	1.67 (0.7)	1.77 (0.8)	0.001
Neutrophils (μ/L)		7.47 (2.62)	8.16 (3.64)	8.08 (3.54)	0.332
Platelet Count (μ/L)		298 (104)	249 (97)	255 (99)	0.13
Mean Platelet Volume (fl)		10 (1.3)	10.5 (1.2)	10.4 (1.2)	0.068
Mortality (%)	Alive	26 (92.9)	164 (80.8)	190 (82.3)	N/A
	Exitus	2 (7.1)	39 (19.2)	41 (17.7)	

sPESI: simplified pulmonary embolism severity index. AST: aspartate aminotransferase, ALT: alanine transaminase. *p* values were calculated by an independent samples test, with variances investigated by Levene’s test for equality and the Mann–Whitney U test, depending on the parametric distribution, when appropriate.

**Table 2:** Pulmonary thromboembolism severity, involvement, hospitalization durations, and ratio comparison.

Parameters (Median, 25th–75th)		sPESI Classification			
		Low-Risk Group (n = 28)	High-Risk Group (n = 203)	Total (n = 231)	*p* Value
PT Severity (%)	Nonmassive	27 (96.4)	187 (92.1)	214 (92.6)	N/A
	Massive	1 (3.6)	16 (7.9)	17 (7.4)	
Pulmonary Arterial Involvement (%)	Unilateral	16 (57.1)	116 (57.1)	132 (57.1)	
	Bilateral	12 (42.9)	87 (42.9)	99 (42.9)	
ICU Admission Requirement (%)	Not Required	24 (85.7)	126 (62.1)	150 (64.9)	
	Required	4 (14.3)	77 (37.9)	81 (35.1)	
Intubation Requirement (%)	No	28 (100)	197 (97)	225 (97.4)	
	Present	0 (0)	6 (3)	6 (2.6)	
ICU Admission Duration (Days)		6 (4–8)	6 (4–10)	6 (4–10)	0.697
Ward Admission Duration (Days)		6 (5–8)	8 (6–11)	8 (6–11)	0.006
Total Hospitalization Duration (Days)		7 (5–10)	10 (6–14)	9 (6–13)	0.007
Neutrophile-to-Lymphocyte Ratio (%)		2.7 (1.7–7.8)	4.9 (3.0–8.5)	4.6 (2.7–8.4)	0.005
Platelet-to-Lymphocyte Ratio (%)		143.3 (77.8–219.3)	156 (113–241)	154.7 (111.5–241.6)	0.233
MPV-to-Lymphocyte Ratio (%)		3.9 (2.8–7.5)	7.1 (4.7–11.0)	7.0 (4.3–10.0)	0.001

sPESI: simplified pulmonary embolism severity index. ICU: intensive care unit. PT: pulmonary thromboembolism. MPV: mean platelet volume. *p* values were calculated by Mann–Whitney U test, due to nonparametric distribution. For parameters defined with N/A, no further analyses were performed due to insufficient patient count for Chi-square test. Pulmonary arterial involvement had the same distribution in risk groups, and no further investigation was performed for these.

**Table 3:** Pulmonary thromboembolism severity and outcome comparison with ratio results.

Parameters (Median, 25th–75th)		NLR	PLR	MPVLR
PT Severity	Nonmassive	4.5 (2.7–8.4)	154.5 (111.5–241.9)	7.0 (4.3–10.4)
	Massive	5.8 (3.5–7.9)	160.3 (114.0–222.4)	7.0 (4.9–8.7)
	*p* Value	0.632	0.958	0.986
ICU Admission Requirement	Not Required	3.8 (2.5–7.4)	145.3 (105.4–207.4)	5.8 (4.0–8.2)
	Required	7.5 (3.9–11.5)	169.7 (113.0–306.8)	8.2 (5.6–12.7)
	*p* Value	0.001	0.001	0.001
Intubation Requirement	No	4.5 (2.7–8.3)	154.5 (110.9–233.9)	6.8 (4.3–9.7)
	Present	9.35 (6.1–12.7)	292.1 (187.2–397.7)	11.85 (8.1–14.5)
	*p* Value	0.025	0.008	0.011
Mortality	Alive	4.3 (2.6–7.8)	146.95 (109.5–222.4)	6.05 (4.1–8.5)
	Exitus	10.5 (5.4–12.8)	187.2 (148.9–341.1)	9.6 (7.5–13.7)
	*p* Value	0.001	0.007	0.001

ICU: intensive care unit, PT: pulmonary thromboembolism, NLR: neutrophile-to-lymphocyte ratio, PLR: platelet-to-lymphocyte ratio, MPVLR: mean-platelet-volume-to-lymphocyte ratio. *p* values were calculated by Mann–Whitney U test, due to nonparametric distribution.

**Table 4:** Correlation analysis between ratios and hospitalization durations.

Correlation Analysis		NLR	PLR	MPVLR
ICU Admission Duration	Correlation Coefficient	0.248	0.212	0.160
	*p* Value	0.025	0.058	0.154
	N	81	81	81
Ward Admission Duration	Correlation Coefficient	0.075	0.069	0.061
	*p* Value	0.285	0.325	0.388
	N	205	205	205
Total Hospitalization Duration	Correlation Coefficient	0.153	0.145	0.098
	*p* Value	0.020	0.028	0.137
	N	231	231	231

ICU: intensive care unit, NLR: neutrophile-to-lymphocyte ratio, PLR: platelet-to-lymphocyte ratio, MPVLR: mean-platelet-volume-to-lymphocyte ratio. Spearman’s rho correlation was used to investigate correlations between nonparametric values.

## Data Availability

Data utilized in this study are available upon reasonable request from the authors.
